# Supplementary material for: Association of Physical and Emotional Parameters with Performance of Firefighters: A Systematic Review
Source: Int J Environ Res Public Health. 2024 Aug 19;21(8):1097. doi: 10.3390/ijerph21081097 (PMC11354647; doi:10.3390/ijerph21081097)
Supplement: Supplementary file 1 [file ijerph-21-01097-s001.zip › File S1 - Search criteria.pdf]

Search criteria

Pubmed

((firefighter OR firefighting OR "fire fighter" OR "fire rescue" OR "public safety")) AND (test OR evaluation OR task OR exercise OR performance OR activity OR emergency OR ability OR simulation OR demands OR physical OR cognitive OR stress OR burnout OR motivation OR wellbeing OR fatigue OR mental OR emotional OR psychologic OR chronic OR recuperation OR rating of perceived exertion OR humor OR respiratory tract infection)

Cochrane

("firefighter" OR "firefighting" OR "fire fighter" OR "fire rescue" OR "public safety"):ti,ab,kw AND ("test" OR "evaluation" OR "task" OR "exercise" OR "performance" OR "activity" OR "emergency" OR "ability" OR "simulation" OR "demands" OR "physical" OR "cognitive" OR "stress" OR "burnout" OR "motivation" OR "wellbeing" OR "fatigue" OR "mental" OR "emotional" OR "psychologic" OR "chronic" OR "recuperation" OR "rating of perceived exertion" OR "humor" OR "respiratory tract infection"):ti,ab,kw

Scopus

((firefighter OR firefighting OR "fire fighter" OR "fire rescue" OR "public safety")) AND (test OR evaluation OR task OR exercise OR performance OR activity OR emergency OR ability OR simulation OR demands OR physical OR cognitive OR stress OR burnout OR motivation OR wellbeing OR fatigue OR mental OR emotional OR psychologic OR chronic OR recuperation OR rating AND of AND perceived AND exertion OR humor OR respiratory AND tract AND infection)

Embase

('firefighter'/exp OR firefighter OR 'firefighting'/exp OR firefighting OR 'fire fighter'/exp OR 'fire fighter' OR 'fire rescue' OR 'public safety'/exp OR 'public safety') AND ('test'/exp OR test OR 'evaluation'/exp OR evaluation OR 'task'/exp OR task OR 'exercise'/exp OR exercise OR

'performance'/exp OR performance OR 'activity'/exp OR activity OR 'emergency'/exp OR emergency OR 'ability'/exp OR ability OR 'simulation'/exp OR simulation OR demands OR physical OR cognitive OR 'stress'/exp OR stress OR 'burnout'/exp OR burnout OR 'motivation'/exp OR motivation OR 'wellbeing'/exp OR wellbeing OR 'fatigue'/exp OR fatigue OR mental OR emotional OR psychologic OR chronic OR recuperation OR 'rating of perceived exertion'/exp OR 'rating of perceived exertion' OR (rating AND of AND perceived AND ('exertion'/exp OR exertion)) OR 'humor'/exp OR humor OR 'respiratory tract infection'/exp OR 'respiratory tract infection' OR (('respiratory'/exp OR respiratory) AND ('tract'/exp OR tract) AND ('infection'/exp OR infection)))

ESPORTDiscus

(("firefighter" OR "firefighting" OR "fire fighter" OR "fire rescue" OR "public safety") AND (("test" OR "evaluation" OR "task" OR "exercise" OR "performance" OR "activity" OR "emergency" OR "ability" OR "simulation" OR "demands" OR "physical" OR "cognitive" OR "stress" OR "burnout" OR "motivation" OR "wellbeing" OR "fatigue" OR "mental" OR "emotional" OR "psychologic" OR "chronic" OR "recuperation" OR "rating of perceived exertion" OR "humor" OR "respiratory tract infection")
