# Supplementary material for: Association of Physical and Emotional Parameters with Performance of Firefighters: A Systematic Review
Source: Int J Environ Res Public Health. 2024 Aug 19;21(8):1097. doi: 10.3390/ijerph21081097 (PMC11354647; doi:10.3390/ijerph21081097)
Supplement: Supplementary file 1 [file ijerph-21-01097-s001.zip › Table S1 - PEDro scale.pdf]

Author	Fitness tests	Performance tests	Performance test results	PEDro scale score
Davis et al., 1982	AF = 5 min step test; Balke treadmill test (Beckman Metabolic Measurement)<br>UBe = chin ups; push-ups (maximum reps)<br>UBs = hand grip strength<br>LBe = N/A<br>LBs = N/A<br>ANc = N/A<br>ANp = standing long jump (m)<br>ABe = sit ups in 2 min (maximum reps)<br>FL = sit and reach<br>BF% = estimated (Zuti and Golding)	1. Ladder extension<br>2. Standpipe carry<br>3. Hose pull<br>4. Simulated rescue<br>5. Simulated forcible entry	1. LE (min): 0.5 ± 0.16<br>2. S (min): 1.6 ± 0.40<br>3. HP (min): 1.0 ± 0.52<br>4. RV (min): 2.4 ± 0.97<br>5. FE (min): 1.5 ± 2.67<br>6. Avarage heart rate (BPM): 168.9 ± 11.81<br>7. TT (min): 7 ± 2.67	4
Myhre et a. 1997	AF = sub-maximal cycle ergometry (estimated from heart rate response)<br>UBe = bench press (80 lb, rate 30/min, maximum reps)<br>UBs = bench press 1RM; upright forearm curl 1RM; upright rowing 1RM;<br>LBe = N/A<br>LBs = leg press 1 RM<br>ANc = N/A<br>ANp = N/A<br>ABe = N/A<br>FL = N/A<br>BF% = hydrostatic weighing	1. Structural search and rescue	1. Structural search and rescue (min:s): 6:17 ± 5:16<br>2. Peak heart rate (bpm): 186.0 ± 11.5<br>3. Ventilation (I.min-1): 104.6 ± 31.3	4

Williford et al. 1999	AF = 1.5 mile run<br>UBe = push-ups, pull-ups (maximum reps)<br>UBs = hand grip strength<br>LBe = N/A<br>LBs = N/A<br>ANc = N/A<br>ANp = N/A<br>ABe = sit-ups (maximum reps in 60 s)<br>FL = sit and reach<br>BF% = skin-caliper (3-site)	1. Stair climb<br>2. Hoisting hose<br>3. Forcible entry<br>4. Hose advance<br>5. Victim rescue	1. SC (s): 53.53 ± 13.68<br>2. HH (s): 32.11 ± 21.87<br>3. FE (s): 30.44 ± 18.62<br>4. HA (s): 19.38 ± 18.88<br>5. RV (s): 48.10 ± 29.36<br>6. TT (s): 303.54 ± 138.13		5

Rhea et al., 2004	AF = 12-min run<br>Ube = row endurance (20.5 kg, maximum reps, dominant hand); bench press endurance (45.5 kg, maximum reps); bicep curl endurance (13.6 kg, maximum reps); seated shoulder press endurance (11.4 kg, maximum reps); hand grip endurance (maintain ≥25.0 kg, s)<br>UBs = bench press 5RM; hand grip strength<br>LBe = squat endurance (61.4 kg, maximum reps)<br>LBs = back squat 5RM;<br>ANc = 400-m run (s)<br>ANp = N/A<br>ABe = ab curls (maximum reps, no time limit, 30 rep per minute cadence)<br>FL = N/A<br>BF% = bodpod	1. Hose Pull<br>2. Stair Climb<br>3. Simulated Victim Drag<br>4. Equipment Hoist	1. HP (s): 21.1 ± 5.6<br>2. SC (s): 85.7 ± 17.1<br>3. RV (s): 37.6 ± 17.1<br>4. EH (s): 17.3 ± 6.9<br>5. TT (s): 161.8 ± 40.8

Perroni et al., 2010	AF = graded incremental treadmill test to exhaustion wearing SCBA (K4b2, Cosmed, Rome, Italy; Accusport Lactate Analyser, Roche, Basel, Switzerland)<br>UBe = N/A<br>UBs = N/A<br>LBe = N/A<br>LBs = N/A<br>ANc = N/A<br>ANp = N/A<br>ABe = N/A<br>FL = N/A<br>BF% = N/A	1. Climb a firemen's ladder and descend a 3-floor building carrying a 20 kg child dummy (child rescue)<br>2. Run for 250 m<br>3. Complete a maze in a dark chamber (find an exit)<br>4. Run for 250 m	1. Child rescue (s): 81.8 ± 25.3<br>2. Run for 250 m (s): 92.8 ± 24.6<br>3. Find an exit (s): 437.5 ± 116.6<br>4. Run for 250 m (s): 91.5 ± 23.7		4
Michaelides et al., 2011	AF = N/A<br>UBe = push-ups (maximum reps)<br>UBs = bench press 1RM; hand grip strength<br>LBe = N/A<br>LBs = back squat 1RM<br>ANc = step test (60 s)<br>ANp = vertical jump<br>Abs* = isometric device (ABMED)<br>ABe = sit-ups (maximum reps in 60 s)<br>FL = sit and reach<br>BF% = bioelectrical Impedance<br><br>*unique study that measured	1. Stair Climb<br>2. Rolled Hose Lift and Move<br>3. Keiser Sled<br>4. Hose Pull and Hydrant Hookup<br>5. Rescue Mannequin Drag<br>6. Charged Hose Advance	1. SC (min): 1.58 ± 0.44<br>2. EC (min): 1.35 ± 0.36<br>3. SH (min): 0.48 ± 0.34<br>4. HP (min): 0.84 ± 0.38<br>5. RV (min): 0.19 ± 0.09<br>6. HA (min): 0.11 ± 0.03<br>7. TT (min): 7.07 6 ± 1.76		4

Schmidt et al., 2012	AF = Léger<br>UBe = bench press (45 Kg, maximum reps); bent-over row (20 kg, dominant hand, maximum reps); bicep curls (14 kg, maximum reps); seated shoulder press (12 kg, maximum reps); hand grip endurance (25 kg, s)<br>UBs = bench press 1RM; hand grip strength<br>LBe = leg press (50% of 1-RM, maximum reps)<br>LBs = deadlift 1 RM; leg press 1 RM<br>ANc = 400-m run (s)<br>ANp = N/A<br>ABe = abdominal curl (maximum reps in two minutes)<br>FL = N/A<br>BF% = Harpenden caliper	1. Hose pull<br>2. Stair climb<br>3. Simulated victim drag<br>4. Simulated ladder raise<br>5. Equipment hoist<br><br>Revised Grinder test:<br>1. Simulated ladder raise<br>2. Hose pull<br>3. Static Jaws-of-Life hold<br>4. Tyre and sledgehammer test<br>5. Stair climb<br>6. Attic Craw<br>7. Simulated victim drag	Revised Grinder test TT (s): 407.75 ± 107.60	5
Siddall et al., 2018	AF = graded uphill running protocol (Cosmed K4 B2)<br>UBe = N/A<br>UBs = N/A<br>LBe = N/A<br>LBs = N/A<br>ANc = N/A<br>ANp = N/A<br>ABe = N/A<br>FL = N/A<br>BF% = bioelectrical impedance	1. Equipment carry<br>2. Casualty evacuation<br>3. Hose run	TT (s): 610 ±79	4

Nazari et al., 2018	AF = Modifed Canadian Aerobic Fitness Test's (mCAFT)<br>UBe = N/A<br>UBs = hand grip strength<br>LBe = N/A<br>LBs = NIOSH lower limb strength<br>ANc = N/A<br>ANp = N/A<br>ABe = N/A<br>FL = N/A<br>BF% = N/A	1. Hose drag<br>2. Stair Climb with a High-Rise Pack	1. HH (s): 59.00 ± 15.00<br>2. SC (s): 59.00 ± 14.50	4
Schmit et al., 2019	AF = 3-Min Step Test HR<br>UBe = N/A<br>UBs = bench press 1 RM; grip strength<br>LBe = N/A<br>LBs = back squat 1 RM<br>ANc = N/A<br>ANp = vertical jump<br>ABe = N/A<br>FL = N/A<br>BF% = N/A	1. Stair Climb with high-rise pack<br>2. Charged Hose Advance<br>3. Victim Rescue Randy	1. SC (s): 73.5 ± 16.7<br>2. HA (s): 7.7 ± 2.1<br>3. RV (s): 15.6 ± 4.9<br>4. TT (s): 96.7 ± 23.0	4

Stevenson et al., 2019	AF = maximal treadmill protocol with portable breath-by-breath gas analyzer<br>UBe = N/A<br>UBs = N/A<br>LBe = N/A<br>LBs = N/A<br>ANc = N/A<br>ANp = N/A<br>ABe = N/A<br>FL = N/A<br>BF% = bioelectrical impedance	1. Equipment carry<br>2. Casualty evacuation<br>3. Hose run	Total time (s): 608 ± 90	4
Lessa et al., 2020	AF = maximum incremental test (Léger)<br>UBe = N/A<br>UBs = N/A<br>LBe = N/A<br>LBs = N/A<br>ANc = 300-m run (s)<br>ANp = N/A<br>ABe = N/A<br>FL = N/A<br>BF% = Cescorf caliper	1. Tower climbing<br>2. Hoisting hose<br>3. 40m run<br>4. Forced entry and rescue<br>5. Use of the hose	TT (s): 175,6 ± 25,5<br>Peak HR (bpm): 175,0 ± 11,0<br>[Lac] (mMol.L-1): 13,6 ± 2,6	4

Saari et al., 2020	AF = N/A<br>UBe = N/A<br>UBs = N/A<br>LBe = N/A<br>LBs = N/A<br>ANc = N/A<br>ANp = N/A<br>ABe = N/A<br>FL = N/A<br>BF% = bioelectric impedance	1. The High-Rise Pack Carry<br>2. Hose Hoist<br>3. Forcible Entry<br>4. Hose Advance<br>5. Victim Rescue	TT (s): younger 115.7 ± 19.6; older 105.5 ± 16.47	4
Skinner et al., 2020	AF = Incremental exercise on a motorized treadmill<br>UBe = push-ups (maximum reps)<br>UBs = bench press 3 RM; grip streght<br>LBe = N/A<br>LBs = leg press 3 RM<br>ANc = anaerobic step test<br>ANp = N/A<br>ABe = abdominal curl (maximum reps)<br>FL = sit and reach<br>BF% = dual energy X-ray absorptiometry	1. Hose drag<br>2. Dummy drag<br>3. Stihl saw hold<br>4. Stair climb<br>5. Simulated ARFF emergency protocol	1. HA (s): 10.0 [8.4–12.0]*<br>2. RV (s): 10.6 ± 2.3<br>3. Stihl saw hold (min): 3.7 ± 1.9<br>4. SC (s): 27.5 ± 4.5<br>5. Simulated ARFF emergency protocol (min): 4.3 [4.1–4.5]*<br><br>*data presented as median [interquartile range]	5

Ras et al., 2023	AF = estimated (age, resting heart rate, body mass)<br>UBe = push-ups (maximum reps in 60 s)<br>UBs = grip strength<br>LBe = N/A<br>LBs = back and leg strength dynamometer<br>ANc = N/A<br>ANp = N/A<br>ABe = sit-ups (maximum reps in 60 s)<br>FL = sit and reach<br>BF% = bioelectric impedance	1. Step-ups<br>2. Charged hose drag and pull<br>3. Forcible entry<br>4. Equipment carry<br>5. Ladder raise and extension<br>6. Rescue drag	TT (s): 369.5 [293.3-488.8]*<br><br>*data presented as median [interquartile range]		4
