# Supplementary material for: Association of Physical and Emotional Parameters with Performance of Firefighters: A Systematic Review
Source: Int J Environ Res Public Health. 2024 Aug 19;21(8):1097. doi: 10.3390/ijerph21081097 (PMC11354647; doi:10.3390/ijerph21081097)
Supplement: Supplementary file 1 [file ijerph-21-01097-s001.zip › Table S2 - Results of fitness tests and firefighting performance tests.pdf]

Table S2 – Results of fitness tests and firefighting performance tests.

Study	Fitness tests	Fitness tests results	Performance tests	Performance test results
Davis et al., 1982	AF = 5 min step test; Balke treadmill test (Beckman Metabolic Measurement)<br>UBe = chin ups; push-ups (maximum reps)<br>UBs = hand grip strength<br>LBe = N/A<br>LBs = N/A<br>ANc = N/A<br>ANp = standing long jump (m)<br>ABe = sit ups in 2 min (maximum reps)<br>FL = sit and reach<br>BF% = estimated (Zuti and Golding)	AF = submaximal minute volume (BTPS) 61.2 ± 14.24; maximal minute volume (L.min) 119.5 ± 21.82; submaximal ventilatory equivalence (LE.LO2-1.min) 29.4 ± 5.46; maximal ventilatory equivalence (LE.LO2-1.min) 36.8 ± 5.94; submaximal oxygen pulse (mlO2.BPM): 14.3 ± 2.38; maximal oxygen pulse (mlO2.BPM) 17.9 ± 3.16; maximal heart rate (BPM) 184.0 ± 10.38; submaximal heart rate (BPM) 146.6 ± 14.41; maximal oxygen uptake (ml.Kg-1.L-1) 39,6 ± 6.42; maximal treadmill grade (Balke protocol) 17.8 ± 3.08; cardiovascular fitness score 242.5 ± 57.46<br>Ube = chin ups (N), 5.5 ± 3.61; push-ups (N) 19.0 ± 7.79<br>Ubs = hand grip strength (Kg), 47.4 ± 5.99<br>Anp = standing long jump (ms), 192.9 ± 25.58<br>Abe = sit ups (N), 36.9 ± 11.67<br>FL = (cm) 23.7 ± 9.14<br><br>measures at rest = blood pressure (mm Hg) 128.6 ± 12.42; pulse pressure (mm Hg) 47.5 ± 9.39; heart rate (BPM) 66.7 ± 10.05	1. Ladder extension<br>2. Standpipe carry<br>3. Hose pull<br>4. Simulated rescue<br>5. Simulated forcible entry	1. LE (min): 0.5 ± 0.16<br>2. S (min): 1.6 ± 0.40<br>3. HP (min): 1.0 ± 0.52<br>4. RV (min): 2.4 ± 0.97<br>5. FE (min): 1.5 ± 2.67<br>6. Average heart rate (BPM): 168.9 ± 11.81<br>7. TT (min): 7 ± 2.67
Myhre et a. 1997	AF = sub-maximal cycle ergometry (estimated from heart rate response)<br>UBe = bench press (80 lb, rate 30/min, maximum reps)<br>UBs = bench press 1RM; upright forearm curl 1RM; upright rowing 1RM;<br>LBe = N/A<br>LBs = leg press 1 RM<br>ANc = N/A<br>ANp = N/A<br>ABe = N/A<br>FL = N/A<br>BF% = hydrostatic weighing	AF = VO2 máx. (mL.Kg-1.min-1): 39.4 ± 9.8<br>UBe = (reps) 29.7 ± 12.8<br>UBs = bench press 1RM (lbs) 171.6 ± 47.1; upright forearm curl 1RM (lbs) 89.3 ± 22.6; upright rowing 1RM (lbs) 98.3 ± 23.7<br>LBs = leg press 1 RM (lbs) 399.6 ± 93.7	1. Structural search and rescue	1. Structural search and rescue (min:s): 6:17 ± 5:16<br>2. Peak heart rate (bpm): 186.0 ± 11.5<br>3. Ventilation (l.min-1): 104.6 ± 31.3
Williford et al. 1999	AF = 1.5 mile run<br>UBe = push-ups, pull-ups (maximum reps)<br>UBs = hand grip strength<br>LBe = N/A<br>LBs = N/A<br>ANc = N/A<br>ANp = N/A<br>ABe = sit-ups (maximum reps in 60 s)<br>FL = sit and reach<br>BF% = skin-caliper (3-site)	AF = (s) 737.60 ± 108.11<br>UBe = pull-ups (reps) 9.03 ± 4.79; push-ups (reps) 41.02 ± 14.08<br>UBs = (kg) 116.75 ± 17.67<br>ABe = 39.88 ± 7.75 (maximum reps in 60 s)<br>FL = (cm) 32.00 ± 8.55<br><br>measures at rest: systolic blood pressure (mmHg) 130 ± 13; diastolic blood pressure (mmHg) 81 ± 7	1. Stair climb<br>2. Hoisting hose<br>3. Forcible entry<br>4. Hose advance<br>5. Victim rescue	1. SC (s): 53.53 ± 13.68<br>2. HH (s): 32.11 ± 21.87<br>3. FE (s): 30.44 ± 18.62<br>4. HA (s): 19.38 ± 18.88<br>5. RV (s): 48.10 ± 29.36<br>6. TT (s): 303.54 ± 138.13

Study	Fitness tests	Fitness tests results	Performance tests	Performance test results
Rhea et al., 2004	AF = 12-min run<br>Ube = row endurance (20.5 kg, maximum reps, dominant hand); bench press endurance (45.5 kg, maximum reps); bicep curl endurance (13.6 kg, maximum reps); seated shoulder press endurance (11.4 kg, maximum reps); hand grip endurance (maintain ≥25.0 kg, s)<br>UBs = bench press 5RM; hand grip strength<br>LBe = squat endurance (61.4 kg, maximum reps)<br>LBs = back squat 5RM;<br>ANc = 400-m run (s)<br>ANp = N/A<br>ABe = ab curls (maximum reps, no time limit, 30 rep per minute cadence)<br>FL = N/A<br>BF% = bodpod	AF = (m) 2,181.0 ± 386.9<br>Ube = row endurance (reps) 35.1 ± 13.8; bench press endurance (reps) 37.9 ± 12.2; bicep curl endurance (reps): 30.3 ± 12.6; shoulder press endurance (reps): 31.1 ± 9.4; hand grip endurance (s): 79.3 ± 30.9<br>UBs = bench press 5RM (kg) 217.6 ± 50.7; hand grip strength (kg) 58.8 ± 11.2<br>LBe = squat endurance (reps) 39.4 ± 29.4<br>LBs = squat 5RM (kg) 298.0 ± 192.9<br>ANc = (s) 80.5 ± 12.6<br>ABe = (reps) 79.5 ± 39.7	1. Hose Pull<br>2. Stair Climb<br>3. Simulated Victim Drag<br>4. Equipment Hoist	1. HP (s): 21.1 ± 5.6<br>2. SC (s): 85.7 ± 17.1<br>3. RV (s): 37.6 ± 17.1<br>4. EH (s): 17.3 ± 6.9<br>5. TT (s): 161.8 ± 40.8
Perroni et al., 2010	AF = graded incremental treadmill test to exhaustion wearing SCBA (K4b2, Cosmed, Rome, Italy; Accusport Lactate Analyser, Roche, Basel, Switzerland)<br>UBe = N/A<br>UBs = N/A<br>LBe = N/A<br>LBs = N/A<br>ANc = N/A<br>ANp = N/A<br>ABe = N/A<br>FL = N/A<br>BF% = N/A	AF = Peak [La] 8.8 ± 2.0 (mMol); VO2peak (mL.kg-1.min-1) 43.1 ± 4.9	1. Climb a firemen's ladder and descend a 3-floor building carrying a 20 kg child dummy (child rescue)<br>2. Run for 250 m<br>3. Complete a maze in a dark chamber (find an exit)<br>4. Run for 250 m	1. Child rescue (s): 81.8 ± 25.3<br>2. Run for 250 m (s): 92.8 ± 24.6<br>3. Find an exit (s): 437.5 ± 116.6<br>4. Run for 250 m (s): 91.5 ± 23.7<br>5. TT (s): 704 ± 135
Michaelides et al., 2011	AF = N/A<br>UBe = push-ups (maximum reps)<br>UBs = bench press 1RM;<br>hand grip strength<br>LBe = N/A<br>LBs = back squat 1RM<br>ANc = step test (60 s)<br>ANp = vertical jump<br>Abs* = isometric device (ABMED)<br>ABe = sit-ups (maximum reps in 60 s)<br>FL = sit and reach<br>BF% = bioelectrical Impedance<br>*unique study that measured abdominal strenght	UBe = (reps) 34 ± 16<br>UBs = bench press 1RM (kg) 101.1 ± 19.78; left grip strength (kg) 57.82 ± 8.42; right grip strength (kg) 60.93 ± 8.62; sum of grip strength (kg) 118.75 ± 16.66<br>LBs = (kg) 130.55 ± 24.55<br>ANc = relative power (W.kg-1) 4.27 ± 1.13; power (W) 403.12 ± 101.42<br>ANp = relative power (W.kg-1) 15.84 ± 1.57; power (W) 1,520.64 ± 267.81<br>Abs = (kg) 34.94 ± 11.00<br>ABe = (reps) 38 ± 11<br>FL = (cm) 47.47 ± 7.81<br>BF% = bioelectrical Impedance	1. Stair Climb<br>2. Rolled Hose Lift and Move<br>3. Keiser Sled<br>4. Hose Pull and Hydrant Hookup<br>5. Rescue Mannequin Drag<br>6. Charged Hose Advance	1. SC (min): 1.58 ± 0.44<br>2. EC (min): 1.35 ± 0.36<br>3. SH (min): 0.48 ± 0.34<br>4. HP (min): 0.84 ± 0.38<br>5. RV (min): 0.19 ± 0.09<br>6. HA (min): 0.11 ± 0.03<br>7. TT (min): 7.07 6 ± 1.76
Schmidt et al., 2012	AF = Léger<br>UBe = bench press (45 Kg, maximum reps); bent-over row (20 kg, dominant hand, maximum reps); bicep curls (14 kg, maximum reps); seated shoulder press (12 kg, maximum reps); hand grip endurance (25 kg, s)<br>UBs = bench press 1RM; hand grip strength	AF = multistage shuttle test (level. stage) 7.33 ± 2.05; predicted VO2max (ml.kg-1.min-1) 37.56 ± 6.99<br>UBe = bench press (reps) 29.48 ± 29.48; bent-over row (reps) 36.21 ± 36.21; bicep curls (reps) 30.79 ± 14.67; shoulder press (reps) 32.40 ± 14.02; hand grip endurance (s) 71.94 ± 21.36<br>UBs = bench press (Kg) 98.33 ± 23.57; hand grip strength (Kg) 55.65 ± 8.75<br>LBe = (reps) 33.77 ± 12.36	1. Hose pull<br>2. Stair climb<br>3. Simulated victim drag<br>4. Simulated ladder raise<br>5. Equipment hoist<br><br>Revised Grinder test:	Revised Grinder test TT (s): 407.75 ± 107.60

Study	Fitness tests	Fitness tests results	Performance tests	Performance test results
	LBe = leg press (50% of 1-RM, maximum reps)<br>LBs = deadlift 1 RM; leg press 1 RM<br>ANc = 400-m run (s)<br>ANp = N/A<br>ABe = abdominal curl (maximum reps in two minutes)<br>FL = N/A<br>BF% = Harpenden caliper	LBs = deadlift 1 RM (Kg) 141.77 ± 22.94; leg press 1 RM (Kg) 337.48 ± 81.23<br>ANc = (s) 83.60 ± 15.03<br>ABe = (reps) 82.46 ± 17.22	1. Simulated ladder raise<br>2. Hose pull<br>3. Static Jaws-of-Life hold<br>4. Tyre and sledgehammer test<br>5. Stair climb<br>6. Attic Craw<br>7. Simulated victim drag	
Siddall et al., 2018	AF = graded uphill running protocol (Cosmed K4 B2)<br>UBe = N/A<br>UBs = N/A<br>LBe = N/A<br>LBs = N/A<br>ANc = N/A<br>ANp = N/A<br>ABe = N/A<br>FL = N/A<br>BF% = bioelectrical impedance	AF = VO2 max (L.min-1) 4.0 ± 0.7; VO2 max (mL.kg-1.min-1) 47.7 ± 9.0	1. Equipment carry<br>2. Casualty evacuation<br>3. Hose run	TT (s): 610 ±79
Nazari et al., 2018	AF = Modifed Canadian Aerobic Fitness Test's (mCAFT)<br>UBe = N/A<br>UBs = hand grip strength<br>LBe = N/A<br>LBs = NIOSH lower limb strength<br>ANc = N/A<br>ANp = N/A<br>ABe = N/A<br>FL = N/A<br>BF% = N/A	AF = VO2max (ml.kg-1.min-1) 40.30 ± 6.25<br>UBs = combined grip strength (kg) 115.85 ± 19.56; grip strength right (kg) 59.38 ± 10.28; grip strength lef (kg) 56.47 ± 10.17<br>LBs = (kg) 138.43 ± 27.63<br><br>measures at rest: resting heart rate (bpm) 73.94 ± 10.66	1. Hose drag<br>2. Stair Climb with a High-Rise Pack	1. HH (s): 59.00 ± 15.00<br>2. SC (s): 59.00 ± 14.50
Schmit et al., 2019	AF = 3-Min Step Test HR<br>UBe = N/A<br>UBs = bench press 1 RM; grip strength<br>LBe = N/A<br>LBs = back squat 1 RM<br>ANc = N/A<br>ANp = vertical jump<br>ABe = N/A<br>FL = N/A<br>BF% = N/A	AF = (%HRmax.) 50.0 ± 10.0<br>UBs = bench press 1 RM (kg.body Mass-1) 1.2 ± 0.3; grip strength (kg) 95.6 ± 13.3<br>LBs = back squat 1 RM (kg.body Mass-1) 1.5 ± 0.4<br>ANp = verical jump (cm) 56.9 ± 10.2; peak power relative to body mass (W.kg-1) 92.7 ± 10.4	1. Stair Climb with high-rise pack<br>2. Charged Hose Advance<br>3. Victim Rescue Randy	1. SC (s): 73.5 ± 16.7<br>2. HA (s): 7.7 ± 2.1<br>3. RV (s): 15.6 ± 4.9<br>4. TT (s): 96.7 ± 23.0
Stevenson et al., 2019	AF = maximal treadmill protocol with portable breath-by-breath gas analyzer<br>UBe = N/A<br>UBs = N/A<br>LBe = N/A<br>LBs = N/A<br>ANc = N/A<br>ANp = N/A<br>ABe = N/A<br>FL = N/A<br>BF% = bioelectrical impedance	AF = VO2 máx. (ml.Kg-1.min-1) 47.8 ± 9.0	1. Equipment carry<br>2. Casualty evacuation<br>3. Hose run	Total time (s): 608 ± 90

Study	Fitness tests	Fitness tests results	Performance tests	Performance test results
Lessa et al., 2020	AF = maximum incremental test (Léger)<br>UBe = N/A<br>UBs = N/A<br>LBe = N/A<br>LBs = N/A<br>ANc = 300-m run (s)<br>ANp = N/A<br>ABe = N/A<br>FL = N/A<br>BF% = Cescorf caliper	AF = HRmax (bpm) 190,0 ± 6,0; VO2max. (ml.kg-1.min-1) 48,2 ± 4,0; peak velocity (km.h-1) 12,6 ± 0,7<br>ANc = HRmax (bpm) 183,0 ± 15,0; total time (s) 66,5 ± 4,2; [Lac] (mMol.L-1) 17,0 ± 1,9; velocity (km.h-1) 16,3 ± 1,0	1. Tower climbing<br>2. Hoisting hose<br>3. 40m run<br>4. Forced entry and rescue<br>5. Use of the hose	TT (s): 175,6 ± 25,5<br>Peak HR (bpm): 175,0 ± 11,0<br>[Lac] (mMol.L-1): 13,6 ± 2,6
Saari et al., 2020	AF = N/A<br>UBe = N/A<br>UBs = N/A<br>LBe = N/A<br>LBs = N/A<br>ANc = N/A<br>ANp = N/A<br>ABe = N/A<br>FL = N/A<br>BF% = bioelectric impedance	Age (y) yunger 31.8 ± 3.42; older 44.65 ± 5.18<br>BF% = yunger 15.94 ± 4.31; older 19.49 ± 4.58<br><br>obs.: Primary goal was not correlate tests, therefore only these two variables were accessed.	1. The High-Rise Pack Carry<br>2. Hose Hoist<br>3. Forcible Entry<br>4. Hose Advance<br>5. Victim Rescue	TT (s): younger 115.7 ± 19.6; older 105.5 ± 16.47
Skinner et al., 2020	AF = Incremental exercise on a motorized treadmill<br>UBe = push-ups (maximum reps)<br>UBs = bench press 3 RM; grip strength<br>LBe = N/A<br>LBs = leg press 3 RM<br>ANc = anaerobic step test<br>ANp = N/A<br>ABe = abdominal curl (maximum reps)<br>FL = sit and reach<br>BF% = dual energy X-ray absorptiometry	AF = VO2max (mL.kg-1.min-1) 49.5 ± 6.9; lactate threshold (%VO2max) 86.1 ± 4.9; speed at lactate threshold (km.h-1) 13.1 [12.0–13.7]*<br>UBe = push-ups (reps) 39.3 ± 14.2<br>UBs = bench press 3 RM (kg) 87.5 [78.1–100.0]*; grip strength (kg) 112.3 ± 16.5<br>LBs = leg press 3 RM (kg) 226.6 ± 35.6<br>ANc = anaerobic step test (max) 63.6 ± 8.4; anaerobic step test (W) 479.1 ± 80.4<br>ABe = (reps) 75.0 [30.3–75.0]*<br>FL = (cm) 40.6 ± 8.2<br><br>*data presented as median [interquartile range]	1. Hose drag<br>2. Dummy drag<br>3. Stihl saw hold<br>4. Stair climb<br>5. Simulated ARFF emergency protocol	1. HA (s): 10.0 [8.4–12.0]*<br>2. RV (s): 10.6 ± 2.3<br>3. Stihl saw hold (min): 3.7 ± 1.9<br>4. SC (s): 27.5 ± 4.5<br>5. Simulated ARFF emergency protocol (min): 4.3 [4.1–4.5]*<br><br>*data presented as median [interquartile range]
Ras et al., 2023	AF = estimated (age, resting heart rate, body mass)<br>UBe = push-ups (maximum reps in 60 s)<br>UBs = grip strength<br>LBe = N/A<br>LBs = back and leg strength dynamometer<br>ANc = N/A<br>ANp = N/A<br>ABe = sit-ups (maximum reps in 60 s)<br>FL = sit and reach<br>BF% = bioelectric impedance	AF = absolute V̇O2max (L.min-1) 3.4 [3.3-3.6]; relative V̇O2max (mL.kg-1.min-1) 42.3 [38.4-46.7]<br>UBe = push-ups (resps) 30.0 [21.3-41.0]<br>UBs = grip strength (Kg) 90.9 [80.0-101.9]<br>LBs = (kg) 118.0 [101.6-135.8]<br>ABe = 30.0 [22.0-36.0]<br>FL = (cm) 44.0 [37.0-50.0]<br><br>obs.: All data presented as median [interquartile range]	1. Step-ups<br>2. Charged hose drag and pull<br>3. Forcible entry<br>4. Equipment carry<br>5. Ladder raise and extension<br>6. Rescue drag	TT (s): 369.5 [293.3-488.8]*<br><br>*data presented as median [interquartile range]
