# Supplementary material for: Association of Physical and Emotional Parameters with Performance of Firefighters: A Systematic Review
Source: Int J Environ Res Public Health. 2024 Aug 19;21(8):1097. doi: 10.3390/ijerph21081097 (PMC11354647; doi:10.3390/ijerph21081097)
Supplement: Supplementary file 1 [file ijerph-21-01097-s001.zip › Table S3 - Outcomes and summary of correlations encountered.pdf]

Table S3 – Outcomes and summary of correlations encountered.

Study	Outcomes	Summary of significant correlations	Fitness tests
Davis et al., 1982	Physical work factor (factor 1) in the six criteria related to twenty measured variables (canonical variable loadings, "L"): maximal treadmill heart rate 0.69; maximal treadmill grade 0.47; submaximal oxygen pulse -0.57; push-ups 0.64; sit-ups 0.62; chin-ups 0.61; standing long jump 0.41; age -0.61 and percent fat -0.44.<br><br>Resistance to fatigue factor (factor 2) in the six criteria related to twenty measured variables (canonical variable loadings, "L"): overall cardiovascular score (-0,53); maximal treadmill grade (-0,49) and lean body weight (-0,46). Significant but lower loadings were also found for age (0,42); percent fat (0,40) and maximal heart rate 0,42).	Variables that were more correlated with performance or had strong prediction power in a multivariate regression<br><br>AF = step test score; submaximal oxygen pulse; maximal heart rate; maximal treadmill grade<br>Ube = chin ups; push ups<br>Ubs = hand grip strength<br>Abe = sit ups<br>Body composition = BF %; lean body mass	AF = 5 min step test; Balke treadmill test (Beckman Metabolic Measurement)<br>UBe = chin ups (maximum reps)<br>UBs = hand grip strength<br>LBe = N/A<br>LBs = N/A<br>ANc = N/A<br>ANp = N/A<br>ABe = sit ups in 2 min<br>FL = sit and reach<br>BF% = estimated (Zuti and Golding)
Myhre et a. 1997	Pearson product moment between TT and variables (r):<br><br>Age: 0.38<br>VO2 max. mL.kg-1.min-1: -0.36<br>VOC max. mL.min-1: -0.33<br>Bench Press: -0.18<br>80-1b Bench Press: -0.17<br>Curl: -0.25<br>Row: -0.37<br>% Body Fat: 0.36<br>Lean Body Mass (LBM): -0.21<br><br>All correlations were significant at P<0.05	AF = VO2 máx.<br>UBe = bench press<br>UBs = bench press; upright forearm curl; upright rowing<br>LBs = (not reported)<br>Body composition = BF %; lean body mass; weight<br>Others = age	AF = sub-maximal cycle ergometry (estimated from heart rate response)<br>UBe = bench press (80 lb, rate 30/min, maximum reps)<br>UBs = bench press 1RM; upright forearm curl 1RM; upright rowing 1RM;<br>LBe = N/A<br>LBs = leg press 1 RM<br>ANc = N/A<br>ANp = N/A<br>ABe = N/A<br>FL = N/A<br>BF% = hydrostatic weighing
Williford et al. 1999	Pearson product moment (r):<br><br>FE HH HA RV SC TT<br>Age (years) 0.01 0.03 0.07 0.14 0.48** 0.19<br>% Fat 0.21* 0.23* 0.12 0.25* 0.52** 0.30**<br>Fat free weight (kg) -0.54** -0.45** -0.36** -0.54** -0.20 -0.47**<br>Pull-ups -0.30** -0.30** -0.30* -0.32** -0.47** -0.38**<br>Push-ups -0.36** -0.35** -0.27** -0.38** -0.47** -0.38**<br>1.5 mile run (s) 0.25* 0.30** 0.10 0.23* 0.56** 0.38**<br>Sit and reach (cm) -0.13 -0.08 -0.06 -0.06 -0.25** -0.15<br>Sit-ups -0.22* -0.22* -0.17 -0.22* -0.41** -0.32**<br>Total grip strength (kg) -0.53** -0.55** -0.41** -0.59** -0.39** -0.54**<br>Weight (kg) -0.40** -0.39** -0.27** -0.38** -0.01 -0.30**<br>Height (cm) -0.43** -0.46** -0.28** -0.32** -0.25* -0.40**<br><br>*p<0.05; **p<0.01	AF = 1.5 mile run<br>UBe = push-ups, pull-ups<br>UBs = hand grip strength<br>ABe = sit-ups<br>Body composition = BF %; lean body mass; weight; height<br>Others = age (correlated only with SC); flexibility (correlated only with SC)	AF = 1.5 mile run<br>UBe = push-ups, pull-ups (maximum reps)<br>UBs = hand grip strength<br>LBe = N/A<br>LBs = N/A<br>ANc = N/A<br>ANp = N/A<br>ABe = sit-ups (maximum reps in 60 s)<br>FL = sit and reach<br>BF% = skin-caliper (3-site)
Rhea et al., 2004	Pearson product moment (r):<br><br>TT: total fitness r = -0.62; bench press strength r = -0.66; hand grip strength r = -0.71; bent-over row endurance r = -0.61; bench press endurance r = -0.73; shoulder press endurance r = -0.71; bicep endurance r = -0.69; squat endurance r = -0.47; 400-m sprint time r = 0.79<br><br>All significant correlations (p≤0.05)	Ube = row endurance; bench press endurance; bicep curl endurance; seated shoulder press endurance<br>UBs = bench press; hand grip strength<br>LBe = squat endurance<br>ANc = 400-m run	AF = 12-min run<br>Ube = row endurance (20.5 kg, maximum reps, dominant hand); bench press endurance (45.5 kg, maximum reps); bicep curl endurance (13.6 kg, maximum reps); seated shoulder press endurance (11.4 kg, maximum reps); hand grip endurance (maintain ≥25.0 kg, s)<br>UBs = bench press 5RM; hand grip strength

Study	Outcomes	Summary of significant correlations	Fitness tests
			LBe = squat endurance<br>(61.4 kg, maximum reps)<br>LBs = back squat 5RM;<br>ANc = 400-m run (s)<br>ANp = N/A<br>ABe = ab curls (maximum<br>reps, no time limit, 30 rep<br>per minute cadence)<br>FL = N/A<br>BF% = bodpod
Perroni et al., 2010	The paper did not put a emphasis on the correlation founded, thus the explanation described in the research is as follows:<br><br>A correlation coefficient of 0.09 (p = 0.72) was found between VO2peak recorded during the treadmill test and time to job completion of the simulated intervention. Furthermore, correlation coefficients ranging from 0.09 to 0.53 emerged between VO2peak and time to complete the different tasks.	AF = not correlated	AF = graded incremental treadmill test to exhaustion wearing SCBA (K4b2, Cosmed, Rome, Italy; Accusport Lactate Analyser, Roche, Basel, Switzerland)<br>UBe = N/A<br>UBs = N/A<br>LBe = N/A<br>LBs = N/A<br>ANc = N/A<br>ANp = N/A<br>ABe = N/A<br>FL = N/A<br>BF% = N/A
Michaelides et al., 2011	Pearson product moment (r):<br><br>TT<br>Abdominal strength, kg: -0.53**<br>Relative power (step test), W.kg-1: -0.40**<br>Push-ups (max reps): -0.27*<br>Sit-ups (reps in a minute): -0.41**<br>1-RM bench press, kg: -0.31**<br>Relative power (vertical jump), W.kg-1: -0.41**<br>Sum grip, kg: -0.16<br>1-RM squat, kg: -0.22<br>RHR, b.min-1: 0.36**<br>Sit and reach, cm: 0.01<br>BMI, kg.m-2: 0.34**<br>Body fat %: 0.57**<br>Body weight, kg: 0.19<br>Age, y: 0.42**<br>Waist, cm: 0.31*<br>Hip, cm: 0.24<br><br>* p < 0.05<br>** p < 0.01	UBe = push-ups<br>UBs = bench press 1RM<br>ANc = step test (60 s)<br>ANp = vertical jump<br>Abs = isometric device (ABMED)<br>ABe = sit-ups<br>Body composition = BF %; BMI; waist<br>Others: age; RHR	AF = N/A<br>UBe = push-ups (maximum<br>reps)<br>UBs = bench press 1RM;<br>hand grip strength<br>LBe = N/A<br>LBs = back squat 1RM<br>ANc = step test (60 s)<br>ANp = vertical jump<br>Abs* = isometric device (ABMED)<br>ABe = sit-ups (maximum<br>reps in 60 s)<br>FL = sit and reach<br>BF% = bioelectrical Impedance<br><br>*unique study that measured abdominal strength

<table>
<tr><th>Study</th><th>Outcomes</th><th>Summary of significant correlations</th><th>Fitness tests</th></tr>
<tr><td>Schmidt et al., 2012</td><td>Pearson product moment (r):
<table>
<tr><th></th><th>HP</th><th>SC</th><th>RV</th><th>EH</th><th>LE</th><th>Revised Grinder</th></tr>
<tr><td>Age</td><td>0.209</td><td>0.178</td><td>0.066</td><td>0.010</td><td>-0.042</td><td>0.209</td></tr>
<tr><td>Body Weight</td><td>-0.170</td><td>-0.003</td><td>-0.486**</td><td>-0.330*</td><td>-0.278</td><td>-0.422**</td></tr>
<tr><td>Height</td><td>-0.237</td><td>-0.243</td><td>-0.568**</td><td>-0.436**</td><td>-0.390**</td><td>-0.620**</td></tr>
<tr><td>Fat %</td><td>0.360*</td><td>0.471**</td><td>0.202</td><td>0.252</td><td>0.178</td><td>0.370**</td></tr>
<tr><td>BMI</td><td>-0.049</td><td>0.161</td><td>-0.221</td><td>-0.113</td><td>-0.094</td><td>-0.092</td></tr>
<tr><td>LMM</td><td>-0.374**</td><td>-0.247</td><td>-0.675**</td><td>-0.505**</td><td>-0.405**</td><td>-0.691**</td></tr>
<tr><td>Bench Press Strength</td><td>-0.411**</td><td>-0.353*</td><td>-0.560**</td><td>-0.523**</td><td>-0.413**</td><td>-0.511**</td></tr>
<tr><td>Deadlift</td><td>-0.397**</td><td>-0.181</td><td>-0.547**</td><td>-0.555**</td><td>-0.358*</td><td>-0.543**</td></tr>
<tr><td>Leg press Strength</td><td>-0.261</td><td>-0.248</td><td>-0.435**</td><td>-0.438**</td><td>-0.262</td><td>-0.395**</td></tr>
<tr><td>Hand Grip Strength Avg</td><td>-0.299*</td><td>-0.228</td><td>-0.521**</td><td>-0.503**</td><td>-0.428**</td><td>-0.573**</td></tr>
<tr><td>Bench Press Endurance</td><td>-0.428**</td><td>-0.367*</td><td>-0.550**</td><td>-0.536**</td><td>-0.502**</td><td>-0.508**</td></tr>
<tr><td>Bent-over Row</td><td>-0.467**</td><td>-0.331*</td><td>-0.508**</td><td>-0.426**</td><td>-0.354*</td><td>-0.535**</td></tr>
<tr><td>Bicep Curl</td><td>-0.553**</td><td>0.060</td><td>-0.447**</td><td>-0.340*</td><td>-0.465**</td><td>-0.431**</td></tr>
<tr><td>Shoulder Press</td><td>-0.422**</td><td>-0.425**</td><td>-0.466**</td><td>-0.492**</td><td>-0.408**</td><td>-0.461**</td></tr>
<tr><td>Leg Press Endurance</td><td>0.032</td><td>-0.159</td><td>0.064</td><td>-0.121</td><td>-0.223</td><td>-0.007</td></tr>
<tr><td>Hand Grip Endurance</td><td>-0.144</td><td>-0.117</td><td>-0.388**</td><td>-0.215</td><td>-0.257</td><td>-0.412**</td></tr>
<tr><td>2min Abdominal Curl</td><td>0.036</td><td>-0.107</td><td>0.243</td><td>0.027</td><td>0.094</td><td>0.069</td></tr>
<tr><td>400m Sprint</td><td>0.555**</td><td>0.363*</td><td>0.350*</td><td>0.358*</td><td>0.367*</td><td>0.500**</td></tr>
<tr><td>Multistage Shuttle Test</td><td>-0.368**</td><td>-0.398**</td><td>-0.305*</td><td>-0.215</td><td>-0.141</td><td>-0.464**</td></tr>
<tr><td>VO2 max</td><td>-0.367*</td><td>-0.399**</td><td>-0.306*</td><td>-0.214</td><td>-0.139</td><td>-0.464**</td></tr>
<tr><td>Overall Fitness</td><td>-0.483**</td><td>-0.380**</td><td>-0.607**</td><td>-0.604**</td><td>-0.479**</td><td>-0.629**</td></tr>
</table>
*. Correlation is significant at the 0.05 level (2-tailed).
**. Correlation is significant at the 0.01 level (2-tailed).</td>
<td>AF = Léger
UBe = bench press; bent-over row; bicep curls; seated shoulder press; hand grip endurance
UBs = bench press 1RM; hand grip strength
LBs = deadlift 1 RM; leg press 1 RM
ANc = 400-m run (s)
Body composition = BF %; body weight; height; LMM
Others: overall fitness</td>
<td>AF = Léger
UBe = bench press (45 Kg, maximum reps); bent-over row (20 kg, dominant hand, maximum reps); bicep curls (14 kg, maximum reps); seated shoulder press (12 kg, maximum reps); hand grip endurance (25 kg, s)
UBs = bench press 1RM; hand grip strength
LBe = leg press (50% of 1-RM, maximum reps)
LBs = deadlift 1 RM; leg press 1 RM
ANc = 400-m run (s)
ANp = N/A
ABe = abdominal curl (maximum reps in two minutes)
FL = N/A
BF% = Harpenden caliper</td></tr>
<tr><td>Siddall et al., 2018</td><td>TT with VO2 max (mL.kg-1.min-1): r = -0.711; r² = 0.506, SEE= ± 56 s (explaining ~18% more of the variance in performance)

TT with VO2 max (L.min-1): r = -0.577; r² = 0.332; SEE= ± 65 s

Prediction models for Firefighting simulation completion time arranged in ascending order of r²:

- 1 VO2 max absolute, body mass: R=0.727, R²=0.528, adjusted R²=0.513, SEE(s)=55
- 2 VO2 max relative, LBM/FM: R=0.745, R²=0.555, adjusted R²=0.541, SEE(s)=54
- 3 VO2 max relative, BF%: R=0.752, R²=0.565, adjusted R²=0.552, SEE(s)=53
- 4 VO2 max absolute, BF%: R=0.762, R²=0.580, adjusted R²=0.567, SEE(s)=52
- 5 VO2 max absolute, FM: R=0.765, R²=0.585, adjusted R²=0.572, SEE(s)=52

obs: The prediction model combining absolute cardiorespiratory capacity (L.min-1) and fat mass explained the greatest variance in performance
and elicited the least random error (R=0.765, R2 =0.585, SEE: ±52 seconds)</td>
<td>AF = VO2 máx.
Body composition = BF %; fat mass
Others = age</td>
<td>AF = graded uphill running protocol (Cosmed K4 B2)
UBe = N/A
UBs = N/A
LBe = N/A
LBs = N/A
ANc = N/A
ANp = N/A
ABe = N/A
FL = N/A
BF% = bioelectrical impedance</td></tr>
<tr><td>Nazari et al., 2018</td><td>Pearson correlation coeficcient (r):
<table>
<tr><th></th><th>HH</th><th>SC</th></tr>
<tr><td>VO2max (ml.kg-1.min-1)</td><td>−0.30**</td><td>−0.31**</td></tr>
<tr><td>NIOSH lower limb strength (kg)</td><td>−0.20**</td><td>0.20</td></tr>
<tr><td>Combined grip strength (kg)</td><td>−0.20**</td><td>0.10</td></tr>
<tr><td>Lef grip strength (kg)</td><td>−0.10**</td><td>0.10</td></tr>
<tr><td>Right grip strength (kg)</td><td>−0.25**</td><td>0.10</td></tr>
</table>
**p < 0.05.</td>
<td>AF = VO2 máx.
UBs = hand grip strength (hose drag)
LBs = NIOSH lower limb strength (hose drag)</td>
<td>AF = Modifed Canadian Aerobic Fitness Test’s (mCAFT)
UBe = N/A
UBs = hand grip strength
LBe = N/A
LBs = NIOSH lower limb strength
ANc = N/A
ANp = N/A
ABe = N/A
FL = N/A
BF% = N/A</td></tr>
</table>

Study	Outcomes	Summary of significant correlations	Fitness tests
Schmit et al., 2019	Pearson product moment (r):<br><br>SC HA RV TT<br>Age 0.27 0.19 0.14 0.25<br>Step Test 0.51* 0.52* 0.38* 0.50*<br>Grip Strength -0.61* -0.49* -0.70* -0.64*<br>1 RM BP/BM (kg/BM) -0.79* -0.79* -0.80* -0.82*<br>1 RM BS/BM (kg/BM) -0.70* -0.52* -0.68* -0.70*<br>Vertical Jump -0.67* -0.49* -0.60* -0.66*<br>Peak Power Relative to BM -0.55* -0.30* -0.42* -0.52*<br><br>*P<0.05	AF = 3-Min Step Test HR<br>UBs = bench press 1 RM; grip strength<br>LBs = back squat 1 RM<br>ANp = vertical jump	AF = 3-Min Step Test HR<br>UBe = N/A<br>UBs = bench press 1 RM; grip strength<br>LBe = N/A<br>LBs = back squat 1 RM<br>ANc = N/A<br>ANp = vertical jump<br>ABe = N/A<br>FL = N/A<br>BF% = N/A
Stevenson et al., 2019	Pearson product moment (r) = -0.73, p=0.01, r²= 0.539	AF = VO2 máx.	AF = maximal treadmill protocol with portable breath-by-breath gas analyzer<br>UBe = N/A<br>UBs = N/A<br>LBe = N/A<br>LBs = N/A<br>ANc = N/A<br>ANp = N/A<br>ABe = N/A<br>FL = N/A<br>BF% = bioelectrical impedance
Lessa et al., 2020	Pearson product moment (r):<br><br>Léger test Performance test (“variable” and “r”)<br>HRmax peak HR 0,45; [Lac] 0,45; TT -0,10<br>Peak velocity peak HR 0,62; [Lac] -0,72*; TT -0,42<br>VO2max peak HR 0,64; [Lac] -0,73*; TT 0,42<br><br>300 m run test Performance test (“variable” and “r”)<br>HR max peak HR 0,15; [Lac] 0,12; TT 0,48<br>TT peak HR -0,15; [Lac] -0,01; TT 0,66<br>[Lac] peak HR 0,46; [Lac] 0,81*; TT -0,52<br><br>*p<0.05	AF = maximum incremental test (VO2 máx. and peak velocity correlates with [Lac] of performance)<br>ANc = 300-m run (s) ([Lac] of test correlates with [Lac] of performance)	AF = maximum incremental test (Léger)<br>UBe = N/A<br>UBs = N/A<br>LBe = N/A<br>LBs = N/A<br>ANc = 300-m run (s)<br>ANp = N/A<br>ABe = N/A<br>FL = N/A<br>BF% = Cescorf caliper
Saari et al., 2020	Pearson product moment (r)<br><br>age: r = 0.297, p = 0.017<br>body fat percentage: r = 0.336, p = 0.065	Others = age	AF = N/A<br>UBe = N/A<br>UBs = N/A<br>LBe = N/A<br>LBs = N/A<br>ANc = N/A<br>ANp = N/A<br>ABe = N/A<br>FL = N/A<br>BF% = bioelectric impedance

<table>
<thead>
<tr><th>Study</th><th>Outcomes</th><th>Summary of significant correlations</th><th>Fitness tests</th></tr>
</thead>
<tbody>
<tr>
<td>Skinner et al., 2020</td>
<td>Correlation coefficients (Pearson’s r) for body composition and physical fitness measures with simulated firefighting emergency task performance.
<table>
<tr><th></th><th>HA</th><th>RV</th><th>Stihl saw hold</th><th>SC</th><th>Simulated ARFF</th></tr>
<tr><td>Age (years)</td><td>0.062</td><td>0.389*</td><td>0.211</td><td>0.312</td><td>0.306</td></tr>
<tr><td>Height (cm)</td><td>0.275</td><td>0.083</td><td>0.082</td><td>0.069</td><td>−0.325*</td></tr>
<tr><td>Body mass (kg)a</td><td>0.048</td><td>−0.069</td><td>0.145</td><td>0.159</td><td>−0.148</td></tr>
<tr><td>BMI (kg m2)</td><td>−0.075</td><td>−0.146</td><td>0.154</td><td>0.185</td><td>0.094</td></tr>
<tr><td>Fat mass (kg)</td><td>0.154</td><td>0.099</td><td>0.129</td><td>0.270</td><td>0.318*</td></tr>
<tr><td>Lean mass (kg)</td><td>−0.065</td><td>−0.102</td><td>0.168</td><td>0.083</td><td>−0.429**</td></tr>
<tr><td>Body fat (%)</td><td>0.125</td><td>0.153</td><td>0.071</td><td>0.215</td><td>0.481**</td></tr>
<tr><td>Arm length (cm)a</td><td>0.428**</td><td>0.273</td><td>0.257</td><td>−0.055</td><td>−0.169</td></tr>
<tr><td>Leg length (cm)</td><td>0.311</td><td>0.238</td><td>0.178</td><td>0.102</td><td>−0.177</td></tr>
<tr><td>3RM bench press (kg)a</td><td>−0.471**</td><td>−0.155</td><td>0.130</td><td>−0.093</td><td>−0.115</td></tr>
<tr><td>3RM leg press (kg)</td><td>−0.156</td><td>−0.304</td><td>−0.192</td><td>0.025</td><td>−0.240</td></tr>
<tr><td>Total grip strength (kg)</td><td>−0.106</td><td>−0.371</td><td>0.205</td><td>−0.025</td><td>−0.189</td></tr>
<tr><td>Anaerobic step test (W)</td><td>−0.099</td><td>−0.305</td><td>−0.003</td><td>−0.277</td><td>−0.549**</td></tr>
<tr><td>Sit and reach (cm)</td><td>−0.234</td><td>−0.249</td><td>−0.084</td><td>−0.051</td><td>−0.161</td></tr>
<tr><td>Abdominal curl (reps)a</td><td>−0.269</td><td>−0.154</td><td>0.029</td><td>−0.190</td><td>−0.288</td></tr>
<tr><td>Push ups (reps)</td><td>−0.385**</td><td>−0.141</td><td>0.090</td><td>−0.288</td><td>−0.126</td></tr>
<tr><td>VO2 max (mL kg-1 min-1)</td><td>0.040</td><td>−0.045</td><td>−0.117</td><td>−0.096</td><td>−0.514**</td></tr>
<tr><td>Lactate threshold (km h-1)</td><td>−0.054</td><td>−0.123</td><td>−0.165</td><td>−0.082</td><td>−0.654**</td></tr>
</table>
BMI, body mass index; LM, lean mass; RM, repetition maximum; max, maximum; W, watts; VO˙ 2 max, maximal aerobic capacity.
a Data presented as Spearman’s rho. * Correlation is significant at the 0.05 level (2-tailed). ** Correlation is significant at the 0.01 level (2-tailed).</td>
<td>AF = VO2 máx.; speed at lactate threshold
ANc = anaerobic step test
Body composition = BF %; height; lean mass; fat mass</td>
<td>AF = Incremental exercise on a motorized treadmill
UBe = push-ups (maximum reps)
UBs = bench press 3 RM; grip streght
LBe = N/A
LBs = leg press 3 RM
ANc = anaerobic step test
ANp = N/A
ABe = abdominal curl (maximum reps)
FL = sit and reach
BF% = dual energy X-ray absorptiometry</td>
</tr>
<tr>
<td>Ras et al., 2023</td>
<td><table>
<tr><th></th><th>β</th><th>p</th></tr>
<tr><td>abV̇O2max (L·min−1)</td><td>−0.159</td><td>0.002**</td></tr>
<tr><td>relV̇O2max (mL·kg−1·min−1)</td><td>−0.016</td><td>0.790</td></tr>
<tr><td>Grip strength (kg)</td><td>−0.263</td><td><0.001**</td></tr>
<tr><td>Leg strength (kg)</td><td>−0.257</td><td><0.001**</td></tr>
<tr><td>Push-ups (rpm)</td><td>−0.339</td><td><0.001**</td></tr>
<tr><td>Sit-ups (rpm)</td><td>−0.315</td><td><0.001**</td></tr>
<tr><td>Sit-and-reach (cm)</td><td>0.94</td><td>0.063</td></tr>
<tr><td>Lean body Mass (kg)</td><td>−0.233</td><td><0.001**</td></tr>
<tr><td>Age (years)§</td><td>0.274</td><td><0.001**</td></tr>
<tr><td>Body mass index (kg·m−2)</td><td>−0.036</td><td>0.477</td></tr>
<tr><td>Bodyfat percentage (%)</td><td>0.051</td><td>0.352</td></tr>
<tr><td>Waist circumference (cm)</td><td>0.050</td><td>0.369</td></tr>
<tr><td>Systolic blood pressure (mmHg)</td><td>−0.49</td><td>0.319</td></tr>
<tr><td>Diastolic blood pressure (mmHg)</td><td>0.035</td><td>0.483</td></tr>
<tr><td>Total cholesterol (mmol·L−1)</td><td>−0.42</td><td>0.405</td></tr>
<tr><td>LDL-C (mmol·L−1)</td><td>−0.038</td><td>0.433</td></tr>
<tr><td>HDL-C (mmol·L−1)</td><td>−0.130</td><td>0.008**</td></tr>
<tr><td>Triglycerides (mmol·L−1)</td><td>0048</td><td>0.334</td></tr>
<tr><td>Non-fasting glucose (mmol·L−1)</td><td>−0.068</td><td>0.156</td></tr>
<tr><td>Diet (score)</td><td>0.064</td><td>0.183</td></tr>
<tr><td>Weekly MET (MET·min−1)¶</td><td>−0.179</td><td><0.001**</td></tr>
<tr><td>Framingham risk score (%)</td><td>0.120</td><td>0.146</td></tr>
<tr><td>Heart rate variability (ms)</td><td>−0.112</td><td>0.021*</td></tr>
<tr><td>SDNN (ms)</td><td>−0.118</td><td>0.021*</td></tr>
<tr><td>RMSSD (ms)</td><td>−0.087</td><td>0.083</td></tr>
<tr><td>LF (Hz)</td><td>−0.030</td><td>0.540</td></tr>
<tr><td>HF (Hz)</td><td>0.033</td><td>0.486</td></tr>
<tr><td>LF/HF (Hz)</td><td>−0.002</td><td>0.965</td></tr>
</table></td>
<td>AF = ab VO2 máx.
UBe = push-ups
UBs = grip strength
LBs = back and leg strength dynamometer
ABe = sit-ups
Body composition = lean body mass
Others = age; HDL-C; weekly MET; heart rate variability; SDNN</td>
<td>AF = estimated (age, resting heart rate, body mass)
UBe = push-ups (maximum reps in 60 s)
UBs = grip strength
LBe = N/A
LBs = back and leg strength dynamometer
ANc = N/A
ANp = N/A
ABe = sit-ups (maximum reps in 60 s)
FL = sit and reach
BF% = bioelectric impedance</td>
</tr>
</tbody>
</table>

Study	Outcomes	Summary of significant correlations	Fitness tests
	*p<0.05; **p <0.01<br><br>LDL-C, low-density lipoprotein cholesterol; HDL-C, high-density lipoprotein cholesterol; SDNN, standard deviation of all normal-to-normal; RMSSD, root-mean-square of successive differences; LF, low-frequency; HF, high frequency; LF/HF, low and high frequency ratio; β, standardized beta coefficient		
